# Tank-binding kinase 1 regulates inflammation and autophagy in glaucoma

**DOI:** 10.3389/fnins.2025.1735403

**Published:** 2026-01-06

**Authors:** Autumn B. Morgan, Denise M. Inman

**Affiliations:** Department of Pharmaceutical Sciences, North Texas Eye Research Institute, UNT Health Fort Worth, Fort Worth, TX, United States

**Keywords:** mitophagy, NF-κB, normal tension glaucoma, optic neuropathy, TNFα

## Abstract

TANK-binding kinase (TBK1) copy number variations have been identified as a monogenic cause of familial normal tension glaucoma (NTG). While elevated intraocular pressure (IOP) is a common risk factor in primary open angle glaucoma (POAG), NTG develops in the absence of IOP elevation. The current available standard of care for glaucoma is lowering IOP, highlighting the need to explore alternative therapeutic mechanisms. The established roles of TBK1 in nuclear factor kappa B (NF-κB) and interferon regulatory factor (IRF) signaling, as well as its involvement in autophagy, suggest it may contribute to glaucomatous neurodegeneration by exacerbating retinal ganglion cell (RGC) stress and optic nerve (ON) damage. This review aims to consolidate current knowledge on the contribution of TBK1 to glaucomatous pathology, focusing on its genetic and molecular roles. By identifying gaps in understanding, this work seeks to guide future research efforts into the mechanisms underlying TBK1’s influence on NTG and to explore therapeutic strategies targeting its pathways.

## Introduction

Glaucoma is a group of optic neuropathies characterized by the progressive loss of retinal ganglion cells (RGCs). Some of the major risk factors include elevated intraocular pressure (IOP), age, race/ethnicity, and family history (McMonnies, 2017). In 2020, glaucoma was the second leading cause of blindness globally for people 50 years and older (Steinmetz et al., 2021). According to the National Eye Institute, primary open angle glaucoma (POAG) is the most common type of glaucoma in the United States. POAG is commonly associated with elevated IOP, which induces mechanical and biochemical stress on the optic nerve (ON), leading to RGC death. Normal tension glaucoma (NTG) is a subset of POAG that results in RGC degeneration despite no IOP elevation, suggesting additional pathological mechanisms contribute to this disease.

Presently, the only available treatment for glaucoma is medication or surgery that lowers IOP. There is evidence that despite the lack of IOP elevation, NTG patients do benefit from these therapies (Salvetat et al., 2023). This paradox highlights the potential sensitivity of NTG patients to normal IOP fluctuations or a lowered threshold of tolerance and suggests mechanical stress may still play a role in this subset (Salvetat et al., 2023). Regardless of IOP level, treatment with IOP-lowering drugs only slows progression of this disease and some patients will experience complete blindness, which underscores the importance of identifying and addressing IOP-independent pathological mechanisms contributing to neurodegeneration in glaucoma (Susanna et al., 2015). Extensive research has revealed factors such as hypoxia/ischemia, mitochondrial or metabolic dysfunction, oxidative stress, neuroinflammation, apoptosis, and more contribute to glaucoma-associated neurodegeneration (Munemasa and Kitaoka, 2013; Zhang et al., 2023). Understanding these complex and multifactorial mechanisms is critical for developing more effective, neuroprotective strategies to prevent vision loss in glaucoma.

Genome wide association studies have revealed numerous genetic alterations contributing to the genetic basis for glaucoma. While POAG is a complex disease involving contributions from multiple genes and environmental interactions, there have been three genes identified that follow Mendelian inheritance patterns (Trivli et al., 2019). These monogenic mutations of POAG are inherited in an autosomal dominant manner, which allows for a significant increase in disease risk if a mutation occurs in one of these genes. The three genes identified were MYOC (myocilin), OPTN (optineurin), and TBK1 (TRAF family member-associated NF-κB activator (TANK) – Binding Kinase 1) (Trivli et al., 2019).

The TBK1 POAG mutation was first identified in 2011 during a genetic linkage study of a family of African American origin that had 10 members diagnosed with NTG (Fingert et al., 2011). This resulted in the identification of a 780 kb duplication on chromosome 12q14 within the genetic locus for NTG (GLC1P) (Fingert et al., 2011). Investigators found TBK1 expression in several relevant areas of glaucoma pathology – retinal ganglion cells, the nerve fiber layer, and microvasculature of the retina (Fingert et al., 2011). The functional effect of this duplication was assessed by mRNA quantification on fibroblast cells obtained from 6 family members within this family (Fingert et al., 2011). Results showed there was an increased mRNA expression of TBK1 that was 1.6-fold-higher than control samples (Fingert et al., 2011). Investigators suggested that this dysregulated expression could result in elevated protein levels or increased kinase activity (Fingert et al., 2016b). This discovery led to more linkage-based analysis that supported this finding with larger population data in families from various ethnic groups including – African American, Caucasian, and those with Australian or Japanese background (Kawase et al., 2012; Ritch et al., 2014; Awadalla et al., 2015; Fingert et al., 2016b). Additionally, one patient was found to have a triplication in the TBK1 gene (Awadalla et al., 2015). Based on these studies, copy number variations (CNV) are associated with 0.4%–1.3% of NTG cases in various populations (Fingert et al., 2016b).

There have been two models developed to investigate the role of TBK1 copy number variations in NTG. Pluripotent stem cells were collected from skin biopsies of POAG patients with TBK1 duplications to produce retinal ganglion cell-like neurons. This study revealed pathological implications of TBK1 duplications in autophagy that will be discussed later (Tucker et al., 2014). Secondly, a transgenic mouse with TBK1 duplications was generated. These mice successfully recapitulated aspects of NTG – progressive loss of retinal ganglion cells despite no IOP elevation (Fingert et al., 2016a). Furthermore, immunohistochemistry revealed predominant localization of TBK1 protein in RGCs, providing compelling evidence of the importance of this protein in glaucomatous pathology (Fingert et al., 2016a). Despite the creation and application of these models, there have been few publications focused on investigating TBK1 copy number variations.

This review seeks to summarize the work done to understand the role of TBK1 in glaucomatous neurodegeneration in hopes of reviewing gaps in our understanding and inspire future investigations focused on understanding the pathological mechanisms of TBK1 copy number variations in NTG.

## TBK1 – structure/function

TBK1 is a serine/threonine kinase belonging to the non-canonical inhibitors of the kappa B kinase (IKK) family. Initially, TBK1 gained attention for the ability to mediate TRAF-associated NF-κB activator (TANK) -associated NF-κB signaling, which led to its identification and characterization in 1999 (Pomerantz and Baltimore, 1999). TBK1 is now most notably known as an important mediator of innate immune defense, inflammation, and autophagy (Yu et al., 2012; Ahmad et al., 2016; Meena et al., 2016; Pourcelot et al., 2016; Oakes et al., 2017; Zhao et al., 2018; Ruyuan et al., 2020; Tooley et al., 2021). Research has also discovered significant involvement in pathways of cellular proliferation/survival, apoptosis, and energy metabolism (Zhao et al., 2018; Runde et al., 2022). As a result, research has been focused on understanding contributions of TBK1 to various human diseases, such as amyotrophic lateral sclerosis (ALS), frontotemporal dementia (FTD), NTG, childhood herpes encephalitis (HSE), autoimmune diseases, cancer, and viral infections (Morton et al., 2008; Kawase et al., 2012; Ritch et al., 2014; Freischmidt et al., 2015; Ahmad et al., 2016; Meena et al., 2016; Oakes et al., 2017; Ruyuan et al., 2020; Medchalmi et al., 2021; Runde et al., 2022).

**FIGURE 1:**
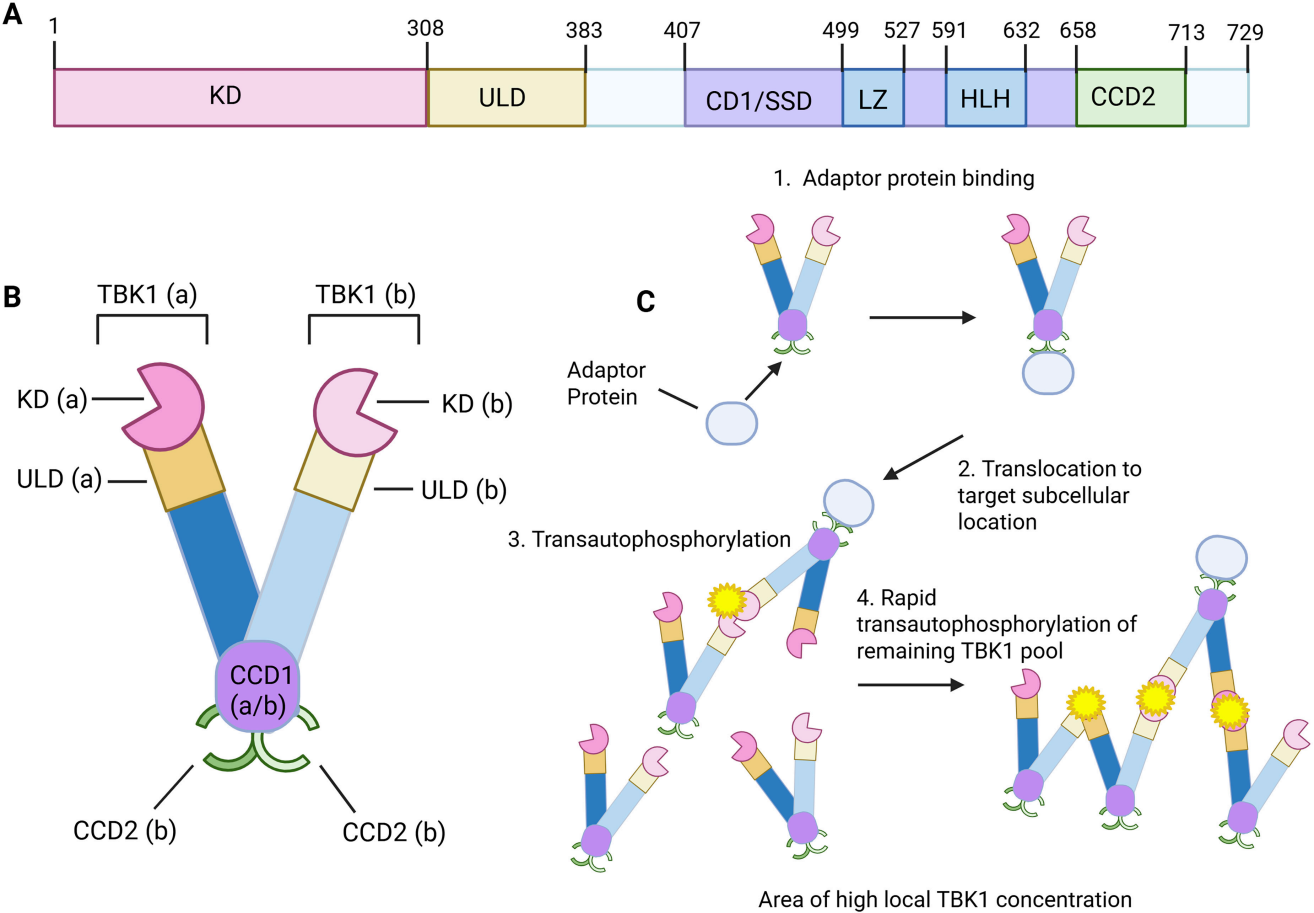
Structure of TBK1 and activation mechanism. **(A)** Representative image of TBK1 protein structure adapted from Ahmad et al. (2016). TBK1 is composed of 729 amino acids and organized into four distinct functional domains – kinase domain (KD), ubiquitin-like domain (ULD), Coiled-coiled domain 1 (CCD1) also known as scaffolding/dimerization domain (SSD1), and coiled-coil domain 2 (CCD2). The CCD1 domain contains leucine zipper motif (LZ) and helix-loop-helix motif (HLH). **(B)** Representative image of TBK1 dimerization structure. Components of the TBK1 structure is labeled with a or b to indicate which monomer the structure belongs to. **(C)** Mechanism of TBK1 activation through autophosphorylation. Depiction inspired by Ma et al. (2012). Adaptor protein binding mediates translocation of TBK1 to subcellular areas of high local TBK1 concentration. This promotes transautophosphorylation of TBK1 that leads to rapid transautophosphorylation of the remaining TBK1 pool. Created in BioRender. Morgan (2025) https://BioRender.com/z03o368.

TBK1 is composed of 729 amino acids and is functionally organized into four distinct domains (Figure 1A). Located at the end of the N-terminus is the kinase domain (KD) responsible for the phosphorylation function (Larabi et al., 2013). Next in line is the ubiquitin-like domain (ULD), which is a regulatory component that controls kinase activity through protein-protein interactions and mediates substrate specificity in collaboration with the coiled coil domain 1 (CCD1) (Larabi et al., 2013). The last two distinct domains are two coiled coil domains (CCD1 and CCD2). Coiled coil domain 1, also known as the scaffolding/dimerization domain (SSD1) contains a leucine zipper (LZ) domain and a helix-loop-helix (HLH) motif (Larabi et al., 2013). These two areas play a role in dimerization of TBK1 and DNA binding. Lastly, at the C terminus is where the coiled coil domain 2 (CCD2) is located, which contains an adaptor-binding motif that allows adaptor proteins to interact, thereby influencing cellular localization and signaling specificity (Larabi et al., 2013).

The most important regulatory controls that determine kinase activation for TBK1 are transautophosphorylation and sub-cellular localization, which work in conjunction with each other (Helgason et al., 2013). The TBK1 homodimer is arranged in a manner that positions the kinase active sites facing away from each other, preventing inappropriate activation (Figure 1B; Ma et al., 2012; Helgason et al., 2013; Larabi et al., 2013). Activation is triggered when adaptor proteins bind to the adaptor binding motif, facilitating translocation to signaling complexes (Figure 1C). These complexes encourage higher local concentrations of TBK1, increasing the likelihood of bringing the kinase activating domain in contact with other dimers, allowing phosphorylation and activation (Helgason et al., 2013). More specifically, the catalytic Asp135 residue contacts and phosphorylates the activation Ser172 residue of a neighboring dimer (Helgason et al., 2013). This activation allows rapid phosphorylation of the remaining TBK1 in the area, forming an activated TBK1 pool (Helgason et al., 2013). This process is critical for activation of TBK1. Additional regulation of TBK1 activity and protein expression is provided by post-translational modifications such as phosphorylation, oligomerization, ubiquitination, SUMOylation, and acetylation (Runde et al., 2022).

## Autophagy

Autophagy is an important mechanism that maintains cellular homeostasis through the degradation and recycling of intracellular components. This process is active at low levels under basal conditions and becomes upregulated in conditions of cellular stress like hypoxia or nutrient deprivation to allow the cell to adapt and survive (Boya et al., 2005; Mazure and Pouysségur, 2010). Autophagy can be non-selective/bulk or selective. Nonselective autophagy involves the indiscriminate engulfment of a large portion of the cytoplasm to recycle material that can be used in metabolic processes to provide energy in response to nutrient deprivation. In contrast, selective autophagy requires recognition machinery to target specific cargo such as mitochondria (mitophagy), protein aggregates (aggrephagy), endoplasmic reticulum (reticulophagy), and pathogens (xenophagy) (Gatica et al., 2018).

**FIGURE 2:**
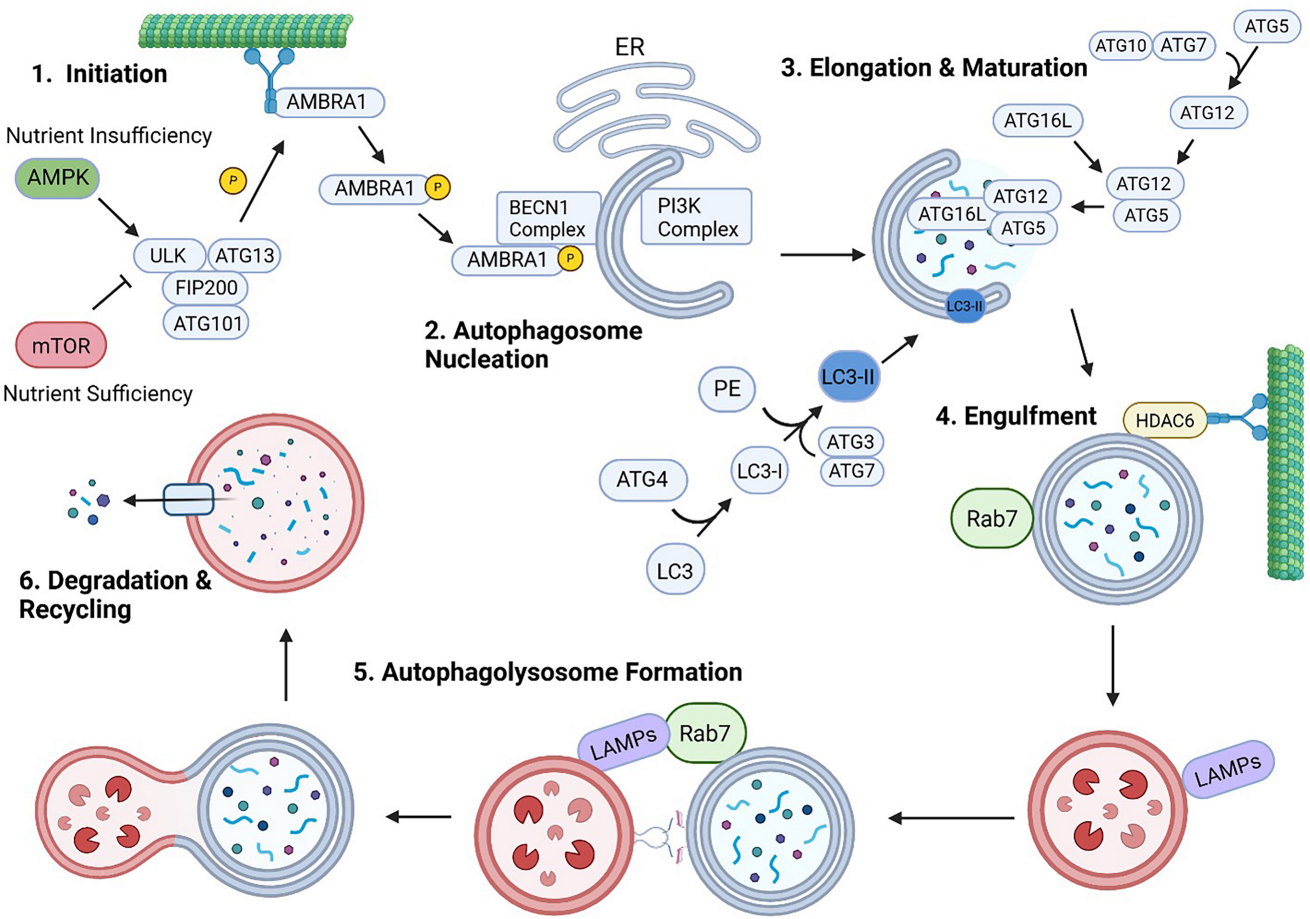
Autophagy mechanism. 1. Autophagy initiation involves activation of ULK1 complex. ULK1 is regulated by AMPK and mTOR, which regulate autophagy in response to cellular energy levels. ULK1 phosphorylates several proteins involved in the process of autophagosome nucleation. Under basal conditions, AMBRA1 is bound to dynein on microtubules. 2. Phosphorylation of AMBRA1 by ULK1 results in translocation of AMBRA1 to the endoplasmic reticulum (ER) where it binds to other complexes (BECN1 complex and PI3K complex) to mediate autophagosome nucleation. 3. The Atg12 and LC3 system undergo conjugation reactions to promote elongation and maturation of the autophagosome. 4. LC3 insertion into the autophagosome allows for recognition and binding to target cargo, while Atg12 system allow elongation of autophagosome membrane to mediate engulfment. 5. Fusion of the autophagosome and lysosome is mediated through the combined action of several proteins (HDAC6, LAMPs, and Rab7). 6. Recycling and degradation of autophagolysosome contents is performed by several acidic hydrolases that were contained in the lysosomal structure. Created in BioRender. Morgan (2025) https://BioRender.com/t25p010.

The process of autophagy involves six stages: initiation, autophagosome nucleation, elongation/autophagosome maturation, engulfment of target cargo, autophagolysosome formation, and degradation/recycling of cargo (Figure 2). Initiation of autophagy in mammalian cells involves the Unc51-like kinase 1 (ULK1) complex, which includes ULK, FIP200 (FAK family kinase-interacting protein of 200 kDa), autophagy-related protein (Atg) 101 and Atg13 (Kroemer et al., 2010). During the initiation step, ULK1 can be activated by AMP-activated protein kinase (AMPK) by phosphorylation of Ser317 and Ser777 during cellular starvation or inhibited by mammalian target of rapamycin (mTOR) phosphorylation at Ser757 during nutrient sufficiency (Figure 2. Step 1) (Jung et al., 2009; Kim et al., 2011). ULK1 phosphorylates several proteins to promote autophagosome biogenesis/nucleation including: Atg4B; Atg9; Atg14L, a subunit of the phosphatidylinositol 3-kinase (PI3K) complex; beclin 1 (BECN1); activating molecule in BECN1-regulated autophagy protein 1 (AMBRA1) (Figure 2. Step 2); as well as other proteins (Di Bartolomeo et al., 2010; Kroemer et al., 2010; Reggiori et al., 2012). For elongation and autophagosome maturation, there are two ubiquitin-like conjugating systems involved – the Atg12 system and the microtubule-associated protein 1 light chain 3 (LC3) system (Figure 2. Step 3). The Atg12 system utilizes Atg7 (an E1-like activating enzyme) and Atg10 (an E2-like activating enzyme) to covalently attach onto Atg5 to form an ATG12-ATG5 complex (Kroemer et al., 2010; Reggiori et al., 2012). This complex then binds to Atg16L to form a scaffold that will facilitate membrane elongation to enclose the targeted cargo (Kroemer et al., 2010; Reggiori et al., 2012). LC3 is synthesized as a precursor protein and undergoes cleavage by Atg4B leaving a glycine residue exposed. This cleaved form is known as LC3-I, the soluble form of the protein. When autophagy is triggered, LC3-I is conjugated to phosphatidylethanolamine (PE) by Atg7 and Atg3 (an E2-like enzyme) (Kroemer et al., 2010; Reggiori et al., 2012). This conjugation creates a lipidated insoluble form, known as LC3-II, that is integrated into the autophagosomes double membrane. This step is essential for membrane elongation and LC3-II is involved in recruiting adaptor proteins that load ubiquitinated (Ub) cargo into the autophagosome. The adaptor proteins contain a ubiquitin-binding domain (UBZ) that allows selective targeting of cargo and an LC3-interacting region (LIR) that allows fusion and engulfment by the autophagosome (Figure 2. Step 4) (Runde et al., 2022). These adaptor proteins include: Optineurin (OPTN), Sequestosome 1 (p62/SQSTM1), nuclear dot protein 52 kDa (NDP52), neighbor of BRCA-1 (NBR1), BCL2/adenovirus E1B 19 kDa protein-interacting protein 3-like (NIX/BNIP3L), and Tax1 binding protein 1 (TAX1BP1) (Runde et al., 2022). The last step in autophagy involves the fusion of the autophagosome with a lysosome, forming an autophagolysosome (Figure 2. Step 5). This structure is where the autophagosome filled with the engulfed contents that will be degraded and recycled. There are a few different proteins involved that are important to mention. HDAC6 (histone deacetylase 6) controls the deacetylation of microtubules to aid in their transport of the autophagosome to the lysosome (Lee et al., 2010; Zhu et al., 2023). SNAREs (soluble N-ethylmaleimide-sensitive factor attachment protein receptor) are responsible for docking and bringing the two membranes in contact to facilitate interactions (Reggiori et al., 2012). Rab7 (Ras-related protein 7) is embedded in the autophagosome membrane and LAMPs (Lysosome-associated membrane proteins) are part of the lysosome membrane (Huynh et al., 2007; Xing et al., 2021). Both of these proteins assist in merging the two membranes. Lastly, the lysosomes contain acidic hydrolases (proteases, lipases, and glycosidases) that degrade proteins, lipids, and other cellular components into simpler molecules that can be recycled (Figure 2. Step 6) (Xu and Ren, 2015).

TBK1 plays several different roles in the process of autophagy. One study investigating the contribution of TBK1 in autophagic microbial defense proposed a role for TBK1 in autophagosome maturation since TBK1 knock-out prevented maturation of autophagosomes into autophagolysosomes (Pilli et al., 2012). The suggested mechanism for this finding was the interaction between TBK1 and Rab8b, an upstream regulator of TBK1, through the colocalization on LC3 of the autophagosome (Pilli et al., 2012). A study by Herhaus et al. (2020), discovered another way TBK1 ensures autophagosome maturation. As described above, an important step in the process of elongation and autophagosome maturation is the formation of LC3-II and insertion into the autophagosome membrane. Atg4 is not only involved in the lipidation of LC3, but it is also responsible for the deconjugation of LC3-II from the autophagosome before lysosome fusion (Agrotis et al., 2019). TBK1 ensures proper autophagosome maturation by phosphorylating LC3-II, which alters the binding affinity for Atg4 and prevents premature removal of LC3-II (Herhaus et al., 2020). Syntaxin 17 is a protein that is thought to be involved in the ULK1 complex since it translocates from the Golgi and colocalizes with FIP200, Atg13, and ULK1 upon induction of autophagy (Kumar et al., 2019). Phosphorylation of Ser202 on Syntaxin 17 by TBK1 was required for the formation of the ULK1 complex during starvation-induced autophagy, revealing an important role for TBK1 in autophagic initiation (Kumar et al., 2019). TBK1 has been found to facilitate autophagosome engulfment by phosphorylation of adaptor proteins, thereby enhancing the binding among ubiquitin cargo, adaptor proteins, and LC3.

Uncontrolled autophagy may be influencing the pathology of glaucoma in patients, as demonstrated in a study utilizing induced pluripotent stem cell (iPSC)- derived retinal cells (Tucker et al., 2014). This study transformed iPSCs from fibroblast samples obtained from NTG patients with TBK1 duplications. Western blot analysis showed a three-fold increase in LC3-II expression in the iPSC-derived RGCs from patients with TBK1 duplications in comparison to the control samples (Tucker et al., 2014), suggesting greater autophagic flux. In a mouse model of acute ocular hypertension, a TBK1 gene knockdown resulted in increased axonal transport and neuroprotection from axon degeneration (Ye et al., 2023). The mechanism of neuroprotection provided by TBK1 knockout was not fully explored, however, it was discovered that TBK1 can inhibit the mammalian target of rapamycin complex 1 (mTORC1) through phosphorylation of Ser1189 on regulatory-associated protein of mammalian target of rapamycin (mTOR) (RAPTOR) (Ye et al., 2023). The investigators mentioned that this preservation of axonal integrity could have been related to mTORC1’s involvement in regulation of protein synthesis that contributes to neuronal development and synaptic plasticity, or its influence in regulation of autophagy (Ye et al., 2023). mTORC1 is capable of suppressing autophagy in response to signals from AMPK regarding cellular energy status, growth factors, or oxygen levels (Rabanal-Ruiz et al., 2017). Additionally, there have been several studies focused on the TBK1 interaction with glaucomatous optineurin mutations that provide further support of dysregulated autophagy due to TBK1. However, this will be discussed in a later section focused on the TBK1 interactions with the OPTN mutations to maintain focus on pathological mechanisms specific to TBK1 alterations.

## Mitophagy

Mitophagy is a specialized form of autophagy that selectively degrades damaged or dysfunctional mitochondria to maintain proper cellular health and homeostasis. This is a crucial aspect of mitochondrial quality control as impaired or damaged mitochondria can generate excessive reactive oxygen species (ROS) and release pro-apoptotic factors, leading to oxidative stress and cell death. Furthermore, mitophagy plays an essential role in tissue that is energy intensive such as brain, heart and retina, where mitochondrial function is crucial for survival. Dysregulation of mitophagy is implicated in various diseases including glaucoma and other neurodegenerative disorders (Wang et al., 2019; Stavropoulos et al., 2023).

Mitophagy is regulated by several key molecular pathways. The PINK1-Parkin pathway is one of the most commonly known. This pathway is crucial in the selective clearance of damaged mitochondria (Narendra et al., 2008). Activation occurs when mitochondria become damaged due to stressors such as oxidative damage or loss of membrane potential (Kataura et al., 2023). Under normal conditions, Phosphatase and Tensin Homolog (PTEN)-induced kinase 1 (PINK1) is continuously imported into healthy mitochondria and rapidly degraded in the mitochondrial inner membrane by proteases (Narendra et al., 2010). When mitochondria become damaged and lose membrane potential, PINK1 accumulation occurs on the outer mitochondrial membrane (OMM) (Narendra et al., 2010). Accumulated PINK1 becomes stabilized and autophosphorylates, activating its kinase function (Rasool et al., 2018). PINK1 phosphorylates ubiquitin molecules on proteins located on the OMM, signaling mitochondrial damage and recruitment of Parkin (Kane et al., 2014). Additionally, PINK1 phosphorylates Parkin (an E3 ubiquitin ligase), activating it (Kim et al., 2008; Kondapalli et al., 2012). Once activated, Parkin translocates to the OMM, where it amplifies the ubiquitination of mitochondrial proteins (Kim et al., 2008; Geisler et al., 2010). This ubiquitination serves as a signal for the recruitment of autophagic machinery, involving binding to autophagy adaptors (NDP52, p62, OPTN, TAX1BP1, and NBR1) which assist autophagosome encapsulation (Geisler et al., 2010; Lazarou et al., 2015; Kataura et al., 2023). Once engulfed, the mitochondria-filled autophagosome fuses with the lysosome and the contents are degraded and recycled.

There are also Parkin-independent pathways of mitophagy. Receptor mediated mitophagy utilizes various outer mitochondrial membrane proteins to directly interact with autophagic machinery like LC3 and gamma-aminobutyric acid receptor-associated protein (GABARAP) through LIRs (Hanna et al., 2012; Rogov et al., 2017). These receptors include BCL2 Interacting Protein 3 (BNIP3), NIP3-like Protein X (NIX/BNIP3L), and FUN14 Domain Containing 1 (FUNDC1). BNIP3, NIX, and FUNDC1 are upregulated during hypoxic conditions to allow adaptation to hypoxic stress (Bellot et al., 2009; Liu et al., 2012). ULK1 can also phosphorylate mitophagy receptors such as FUNDC1 and promote interaction with LC3 (Wu et al., 2014). This is an important process for initiating mitophagy during nutrient deprivation or mitochondrial stress. Lastly, AMBRA1 facilitates mitochondrial clearance by interacting with LC3 in response to mitochondrial stress (Strappazzon et al., 2015).

During PINK-Parkin mediated mitophagy, p62 accumulation was observed on the mitochondria (Matsumoto et al., 2015). It was discovered that phosphorylation of Ser403 on p62/SQSTM1 was required for autophagosome engulfment of mitochondria because mitochondria without the phosphorylated p62 were not engulfed (Matsumoto et al., 2015). TBK1 is responsible for this phosphorylation – indicating the importance of TBK1 regulation in autophagosomal engulfment of mitochondria (Matsumoto et al., 2015). Phosphorylation of Ser72 on Rab7a by TBK1 promoted recruitment of Atg9 vesicles to damaged mitochondria, suggesting an interesting role of TBK1 in autophagosome biogenesis during Parkin-mediated mitophagy (Heo et al., 2018). The collaboration between optineurin and TBK1 in the process of mitophagy has been investigated by several groups due to their involvement in neurodegenerative diseases. During mitophagy, it appears that TBK1 requires recruitment by OPTN for activation since OPTN deletion inhibited TBK1 autophosphorylation (Yamano et al., 2024). Optineurin seems to be equally as dependent on TBK1 during mitophagy. Deletion of TBK1 appeared to prevent localization of OPTN at the autophagosome formation site during Parkin-mediated mitophagy (Yamano et al., 2024). A different study found that TBK1 phosphorylation was not required for recruitment of OPTN to damaged mitochondria, but it was required to stabilize OPTN on mitochondria to allow LC3 recruitment (Moore and Holzbaur, 2016). Additionally, TBK1-dependent phosphorylation of Ser473 and Ser513 on OPTN in HeLa cells enhanced ubiquitin chain binding (Heo et al., 2015). Since it appears that Parkin-mediated autophagy recruits several autophagy receptors (p62/SQSTM1, NDP52, OPTN, and TAX1BP1), the activation and localization of TBK1 by OPTN may be the important initiation step that allows TBK1 access to enhance the binding affinities of the other receptors leading to mitophagic efficiency (Heo et al., 2015; Matsumoto et al., 2015; Moore and Holzbaur, 2016; Richter et al., 2016; Yamano et al., 2024).

Despite the evidence indicating TBK1 heavily influences the mitophagic process, there have been no published studies investigating the pathological effects of TBK1 in NTG with regard to mitophagy. It is possible that TBK1 duplications could promote more mitophagy or maintain mitophagic function. Increased mitophagy could be beneficial. There is ample evidence suggesting metabolic vulnerability and oxidative stress are increased in glaucomatous pathology as a result of dysfunctional mitochondria (Tezel, 2006; Zhang et al., 2023). Unfortunately, mitophagic machinery also seems to be impaired (Stavropoulos et al., 2023). In fact, mitophagy has been investigated as a protective mechanism in several studies (Dai et al., 2018; Hass and Barnstable, 2019). With this in mind, it would be important to investigate the contributions of TBK1 copy number variations to mitophagy in NTG.

While there are no formal publications on mitophagy, one published study analyzed the effects of TBK1 on mitochondrial biogenesis using human stem cell differentiated RGCs with an E50K OPTN mutation that induces NTG (Surma et al., 2023). Results of this study revealed that TBK1 inhibition by BX795 promoted an increase in mitochondrial biogenesis through the activation of the AMPK-PGC1α pathway (Surma et al., 2023). TBK1 inhibition also resulted in decreased apoptosis and increased spare respiratory capacity (Surma et al., 2023). In mouse adipose tissue, TBK1 was shown to inhibit AMPK leading to a decrease in energy expenditure, inflammation, and obesity (Zhao et al., 2018). Since it is possible TBK1 overexpression in NTG would suppress mitochondrial biogenesis and promote or maintain mitophagy, it would be relevant to explore these mechanisms further. Mitochondrial biogenesis and mitophagy are two important mechanisms that provide quality control regulation for cellular mitochondria (Palikaras et al., 2015). This cooperative effort ensures the cell is provided with an adequate supply of functional mitochondria to support the energetic needs of the cell (Palikaras et al., 2015). If there is an imbalance in these two quality control mechanisms, it could result in an overall decrease in mitochondrial numbers and lead to the inability to produce enough energy for cellular survival.

## Inflammation & apoptosis

**FIGURE 3:**
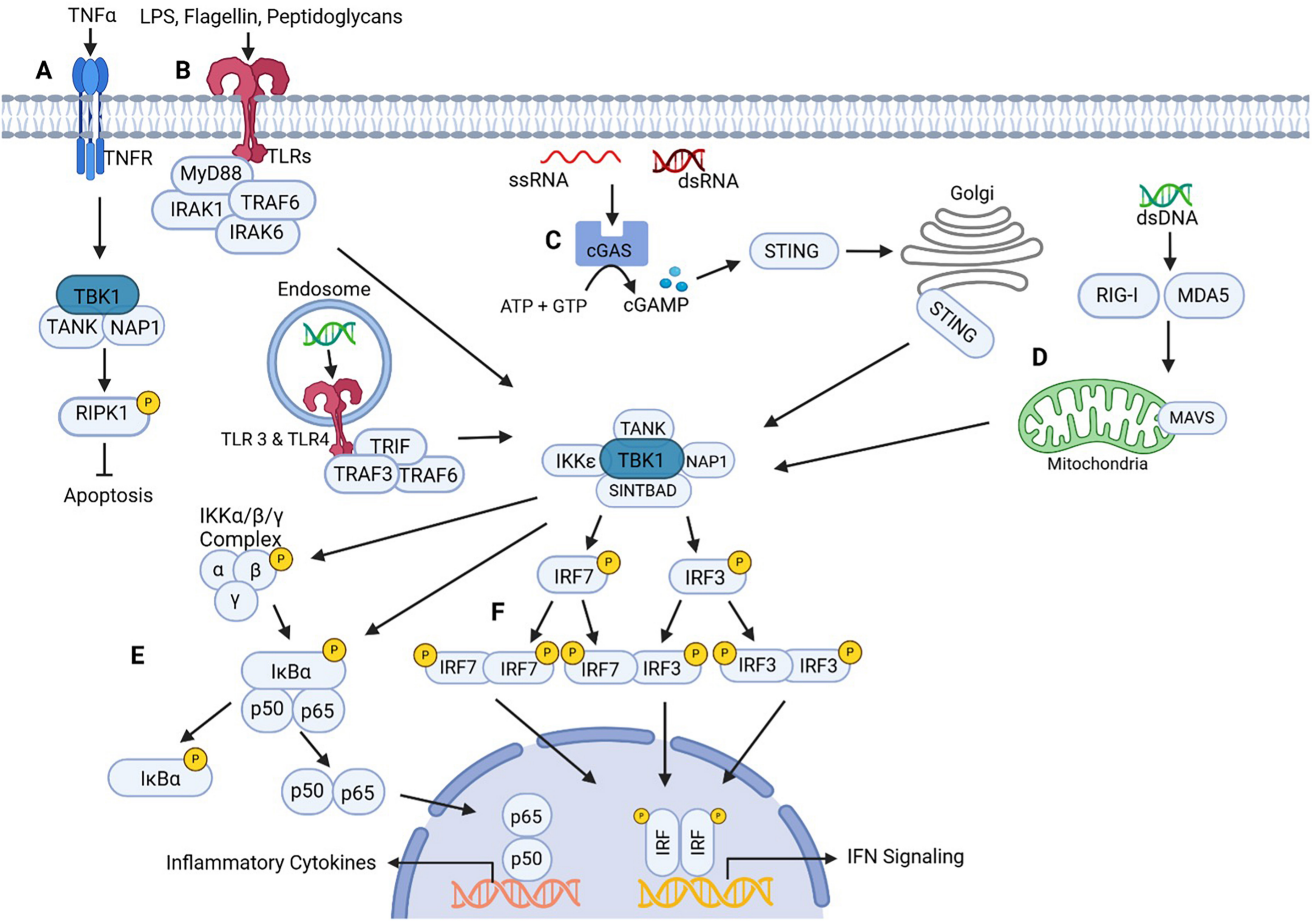
TBK1 inflammatory pathways. **(A)** TBK1 mediated inhibition of TNFα-mediated cell death. Activation of TBK1 by TNFR results in phosphorylation of RIPK1. RIPK1 inhibition stops TNFα induced apoptosis. **(B)** Cell surface TLR activation by pathogen associated molecular patterns (PAMPs) or endosomal TLR activation by nucleic acids leads to recruitment of adapter molecules (MyD88, IRAK1/6, TRAF3/6, and TRIF) that mediate TBK1 activation. **(C)** cGAS activation occurs by recognition of cytosolic RNA. This leads to the synthesis of cGAMP from ATP and GPT, which acts as a signaling molecule for STING. STING is translocated to the golgi apparatus in response to cGAMP signals where it mediates activation of TBK1. **(D)** RLRs detect cytosolic nucleic acids and trigger MAVs oligomerization on mitochondria. This recruits TRAF2, TRAF5, and TRAF6 to synthesize polyubiquitin chains. Polyubiquitination attracts adaptor proteins that mediate TBK1 activation (TANK1, NAP1, TBKBP1, and SINTBAD). **(E)** TBK1 mediates phosphorylation of IKKβ or IκB leading to the dissociation from NF-κB. This allows NF-κB to translocate to the nucleus to transcribe pro-inflammatory cytokines. **(F)** TBK1 phosphorylates IRF3 and/or IRF7, which allows for homo- or hetero- dimerization of IRF. Dimerization leads to IRF translocation to the nucleus where it binds to transcription factors that will upregulate expression of Type-1 IFNs (IFNα or IFNβ). Created in BioRender. Morgan (2025) https://BioRender.com/wli35fw.

TBK1 is a key regulator at the intersection of inflammation and apoptosis, playing a vital role in controlling both immune response and programmed cell death. Inflammation is mediated through activation of NF-κB or interferon regulatory factor (IRF) (Figure 3). There are a number of different pattern recognition receptors (PRRs) involved in the activation of inflammatory pathways mediated by TBK1 including: Toll-like receptors (TLRs), retinoic acid-inducible gene I (RIG-I)-like receptors (RLRs), and cytosolic DNA sensors (CDS).

TLRs are divided into two groups – cell surface TLRs or Endosomal TLRs. Cell surface TLRs are activated upon recognition of components associated with pathogen membranes (e.g., bacterial lipoproteins, peptidoglycans, flagellin, LPS, etc.) (Kawasaki and Kawai, 2014; Li and Wu, 2021). In contrast, endosomal TLRs detect cytosolic nucleic acids [e.g., viral double stranded ribonucleic acids (dsRNA) and single-stranded (ss) RNA] (Kawasaki and Kawai, 2014; Li and Wu, 2021). Activation of TLRs induces dimerization and conformational changes that recruit adaptor molecules to facilitate downstream signaling that will lead to the activation of TBK1 (Figure 3B). Adaptor molecules involved in the downstream signaling are dependent on which TLR is activated, but could include involvement from myeloid differentiation factor 88 (MyD88), TNF receptor-associated factor 3 (TRAF3), TIR-domain-containing adaptor-inducing interferon-β (TRAF) 3 or TRAF6 (Kawasaki and Kawai, 2014; Li and Wu, 2021). Interestingly, TBK1 is stimulated in a TRIF-TRAF3 signaling cascade only when endosomal TLR3 and TLR4 are activated (Zhou et al., 2020).

Two RLRs – RIG-I and melanoma differentiation-associated protein 5 (MDA5) – activate TBK1 through a pathway involving a protein on the outer mitochondrial membrane known as the mitochondrial antiviral signaling protein (MAVS) (Figure 3D). RIG-I and MDA5 detect cytosolic non-self RNA, which induces a conformational change that allows them to interact with MAVS on the mitochondria (Rehwinkel and Gack, 2020). Interaction leads to MAVS oligomerization that recruits TRAF2, TRAF5, and TRAF6 to synthesize poly-ubiquitin chains (Rehwinkel and Gack, 2020). The poly-ubiquitin chains recruit TRAF-associated NF-κB activator 1 (TANK1), NAK-associated protein 1 (NAP1), and TBK1 Binding Protein 1 (TBKBP1) – also known as Similar to NAP1 TBK1 adaptor domain-containing protein (SINTBAD). This will activate TBK1 in this pathway.

Lastly, the cytosolic DNA sensors (CDS) involved in TBK1 activation include cyclic GMP-AMP (cGAMP) synthase (cGAS), DNA-dependent activator of IRFs (DAIs), and DEAD-Box Helicase 41 (DDX41) (Takaoka et al., 2007; Soulat et al., 2008; Xia et al., 2016). The most commonly known pathway involving CDS is known as the cGAS-STING pathway (Figure 3C). cGAS recognizes foreign double-stranded deoxyribonucleic acid (dsDNA) and induces a conformational change (Xia et al., 2016). The conformational change in cGAS opens a catalytic pocket for adenosine triphosphate (ATP) and guanosine triphosphate (GTP) that mediates the synthesis of cGAMP (Xia et al., 2016). cGAMP activates stimulator of interferon genes (STING), which translocates to the Golgi and activates TBK1 (Sun et al., 2013; Pourcelot et al., 2016; Zhang et al., 2019).

TBK1 may be activated in signaling complexes that involve interaction with TANK1, NAP1, SINTBAD/TBKBP1, and IKKε (Peters and Maniatis, 2001; Chariot et al., 2002; Fujita et al., 2003). Once activated, TBK1 will either mediate IFN production through interaction with IRF3/7 (Figure 3F) or production of inflammatory cytokines through NF-κB signaling (Figure 3E). TBK1 will induce homo- or hetero- dimerization of IRF3/7, allowing their translocation to the nucleus, and induction of IFNα/β (Fitzgerald et al., 2003; Sharma et al., 2003; Hiscott, 2007). Alternatively, TBK1 works cooperatively with IKKε to phosphorylate IκBα or independently to phosphorylate IKKβ, RelA/p65, and c-Rel to mediate NF-κB transcription of proinflammatory genes such as interleukin-6 (IL-6), interleukin-1β (IL-1β), and tumor necrosis factor α (TNFα) (Yu et al., 2012; Liu et al., 2017).

Since TBK1 phosphorylates IRF3/7 and NF-κB, it plays a central role in innate immune signaling. Macrophages are important immune cells that monitor the tissue environment for infection or damage, engulf pathogens, and produce mediators that affect downstream signaling pathways. Depending on the physiological conditions, macrophages can differentiate into two subtypes: M1 or M2 (Liu et al., 2017). M1 macrophages are known to produce pro-inflammatory cytokines such as IL-1, IL-6, TNFα, IL-12, and various chemokines; they also support differentiation of inflammatory T cells (Th1 and Th17) (Liu et al., 2017). The M2 macrophage subtype produces anti-inflammatory cytokines, IL-10 and IL-13, playing an important role in the resolution of inflammation and promotion of wound healing (Liu et al., 2017). TBK1 is involved in inflammatory macrophage signaling activated through TLR3 and TLR4 (Yu et al., 2012). Furthermore, TBK1 activated through TLR3 signaling produces proinflammatory and antiviral cytokines [IFNα, IFNβ, and regulated and normal T cell expressed and secreted (RANTES)] in T cells (Yu et al., 2012). Similarly to macrophages, microglia, the resident immune cells of the central nervous system (CNS), have pro- and anti-inflammatory subtypes (Anilkumar and Wright-Jin, 2024). However, NF-κB activation primarily induces a pro-inflammatory microglial state in the CNS (Anilkumar and Wright-Jin, 2024). Additionally, astrocytic activation of NF-κB is associated with elevated brain inflammation and worsening pathological outcomes (Anilkumar and Wright-Jin, 2024). These represent important mechanisms by which prolonged activation of TBK1 signaling could contribute to neuroinflammation.

In murine models of glaucoma, there was significant activation of cGAS-STING signaling in retinal microglia, contributing neuroinflammation and promoting RGC loss (Liu et al., 2024). The proposed mechanism is that retinal tissue injuries, such as mechanical stress induced by IOP or chemical stress like oxidative damage, releases dsDNA within the microglial cytoplasm. The dsDNA acts as a damage-associated molecular pattern (DAMP) that triggers the activation of the cGAS-STING pathway. When cGAS detects the dsDNA, it binds and activates STING on the endoplasmic reticulum. STING mediates the activation of TBK1, which leads to the production of IFN-I and inflammatory cytokines. This induces harmful and widespread microglial activation can drive neuroinflammation and apoptosis in RGCs. In support of this proposed mechanism, intraocular injection of a TBK1 inhibitor, anti-IFNαR1 neutralizing antibody, or deletion of STING either globally or only in microglia, was able to protect RGCs and prevent vision loss (Liu et al., 2024). TBK1 may also be contributing to retinal inflammation and RGC apoptosis by mediating the degradation of proteins involved in transport of proteins that suppress microglial activation. Annexin A1 (ANXA1) is a cytoplasmic protein that can be secreted extracellularly to bind formyl peptide receptors (FPRs) to suppress microglial activation and prevent release of pro-inflammatory cytokines (Parente and Solito, 2004; Li et al., 2018). Additionally, ANXA1 is capable of inducing apoptosis through nuclear translocation that allows direct interaction with *Bid*, a pro-apoptotic gene, during cellular stress (Parente and Solito, 2004). Extracellular secretion of ANXA1 is dependent on transport across the cell membrane by ATP-binding cassette transporter A1 (ABCA1) a transmembrane lipid/protein transporter (Li et al., 2018). Thus, the actions of ABCA1 in determining the location of ANXA1, whether it will accumulate in the cytoplasm or extracellular space, is an important factor in determination of cell fate. In mice with the ischemic-reperfusion model of glaucomatous damage, there was increased expression of TBK1 and decreased expression of ABCA1 (Li et al., 2018). Since mRNA levels of ABCA1 remained stable despite decreased protein expression, it raised the possibility that degradation of the protein may be occurring (Li et al., 2018), leading to the discovery that TBK1 overexpression mediated an increase in ubiquitin-proteasome binding and ubiquitin-proteasome phosphorylation of ABCA1 (Li et al., 2018). A proteasome inhibitor, MG132, prevented the degradation of ABCA1, which further supported the idea that TBK1 was influencing ABCA1 degradation (Li et al., 2018). With this in mind, the increased degradation of ABCA1 prevents the secretion of ANXA1 leading to cytoplasmic accumulation. This would allow ANXA1 to be translocated to the nucleus during glaucomatous stress leading to RGC apoptosis. In contrast, TBK1 could inhibit a mechanism involving a protein associated with inflammation and apoptosis called receptor-interacting serine/threonine-protein kinase 1 (RIPK1) (Figure 3A). Tumor Necrosis Factor Receptor 1 (TNFR1) is involved in inflammation due to the ability to initiate NF-κB signaling that upregulates inflammatory cytokines and it also plays a role as a “death receptor” in apoptosis. RIPK1 appears to be a protein involved in the signaling pathway of TNFR1. Researchers investigating the embryonic lethality of *Tbk1*^/^ and the neuroinflammation produced by TBK1 reduction involved in multiple neurodegenerative diseases discovered that TBK1 can directly phosphorylate T189/T190, which inhibits RIPK1 in murine species and humans (Xu et al., 2018). It appears that TBK1 deficiency sensitizes cells to RIPK1-dependent apoptosis upon stimulation of TNFR (Xu et al., 2018). These results were supported in another study specifically focused on how RIPK1 inhibition by TBK1 can lead to reduced RGC apoptosis in the optic nerve crush model of glaucoma (Ren et al., 2024). When TBK1 was overexpressed using a lentivirus vector, there were decreased levels of inflammatory factors related to the RIPK1 apoptotic signaling pathway, including TNF-α, IL-6 and IL-1β. They also confirmed that TBK1 overexpression decreased expression of RIPK1 (Ren et al., 2024). Additionally, in ischemic retinal injury, TBK1 showed increased activation that led to RGC senescence through direct phosphorylation of Akt on Ser473, which resulted in a downregulation of B lymphoma Mo-MLV insertion region 1 (Bmi) (Li et al., 2017). Bmi is involved in inhibition of senescence associated pathways, such as p16INK4a/p16 and p21 (Li et al., 2017). RGC senescence has been shown to elevate rates of RGC death, though the mechanism is still not fully understood (Li et al., 2017). However, senescent cells have been shown to adopt a secretory phenotype known as senescence-associated secretory phenotype (SASP), which leads to the secretion of pro-inflammatory factors (Muñoz-Espín and Serrano, 2014). There is a possibility that this may be contributing to chronic inflammation and death of RGCs in glaucoma. These studies demonstrate how TBK1 can modulate pathways of apoptosis and inflammation in different ways. Based on these results, TBK1 could be either inhibiting or increasing inflammation and apoptosis in glaucoma. Further investigations need to be done to understand how these different signaling pathways may be working in conjunction to mediate the pathogenesis of NTG.

## Optineurin mutations & TBK1

While this review is focused on TBK1, the discussion would be incomplete without mentioning how TBK1 contributes to the pathogenesis of optineurin mutations in NTG. Three missense mutations in OPTN have been indicated in 16.7% of cases with POAG – glutamine 50 to lysine (E50K), methionine 98 to lysine (M98K), and arginine 545 to glutamine (R545Q) (Rezaie et al., 2002). Studies focused on understanding the contribution of these mutations to glaucoma have revealed that some of these mutations, E50K and M98K specifically, result in modified interactions with TBK1 that influence the pathogenic activity of OPTN.

There are two thoughts on how the E50K mutation contributes to POAG – decreased regulation of NF-κB-dependent gene transcription and/or abnormal activation of TBK1. A study by Zhu et al. (2007), proposed that OPTN may serve as a negative regulator of TNFα induced- NF-κB activation because when miRNA was used to significantly reduce OPTN expression, there were increased levels of NF-κB-dependent gene transcription. Since another study found that dermal fibroblast cultures acquired from a POAG patient with the E50K mutation expressed lower levels of OPTN in comparison to normal patients (Rezaie et al., 2002), the idea that it may be the reduced expression of OPTN and therefore increased activation of TNFα stimulated NF-κB that leads to NTG was supported (Zhu et al., 2007). These investigators also observed that optineurin and NF-κB essential modulator (NEMO) compete when binding to K63-polyubiquitinated RIP in the signaling complex initiated by TNFα and proposed this as the mechanism that negatively regulates this pathway of NF-κB activation (Zhu et al., 2007). Another potential mechanism for the E50K mutation was revealed in a study by Morton et al. (2008), which suggested that abnormal activation of TBK1 is responsible. They not only demonstrated that the E50K mutant OPTN had a tighter binding affinity with TBK1, but also discovered a binding site for OPTN on TBK1 (Morton et al., 2008). Deletion of the 40 residues on the C terminus of TBK1 prevents interaction with TANK and eliminates the ability to interact with OPTN, thereby indicating the critical binding site for OPTN must be located on the C terminal region (Morton et al., 2008). In addition to this, it was shown that the enhanced interaction of OPTN – TBK1 due to the E50K mutation led to prevention of proper oligomerization, resulting in insolubility of this complex. The aggregation of this protein complex could contribute to the ER stress that has been observed in POAG, especially since this mutation has also been linked to autophagic dysfunction – preventing the clearance of this build -up (Chen et al., 2023).

Two studies performed on the RGC-5 cell line explored the contribution of the M98K Optineurin mutation in cell death. The first study focused on the role of Transferrin receptor 1 (TFRC), a protein that facilitates cellular iron uptake, which is important for various cellular functions. The study revealed that the M98K mutation accelerated degradation of TFRC through the lysosomal pathway in the RGC-5 cells (Sirohi et al., 2013). The reduced expression of TFRC would lead to impaired iron import, thereby disrupting iron homeostasis in these cells. Since iron plays a crucial role in the electron transport chain, iron deficiency could lead to mitochondrial dysfunction, decreased energy production, and oxidative stress – leading to cell death. Therefore, they suspected that the disruption in neuronal iron intake may explain the death of cells with E98K-OPTN mutations. This study also suggested that the TFRC degradation occurred through recruitment of RAB12 to autophagosomes since knock-down of RAB12 resulted in increased TFRC expression and decreased RGC-5 cell death (Sirohi et al., 2013). A follow-up study focused on understanding what would trigger this degradation of TFRC through autophagy. It was discovered that the induction of autophagy and cellular death required TBK1 to phosphorylate the E98K-OPTN mutant at Ser177 and that the mutant had increased phosphorylation when compared to the wild type OPTN (Sirohi et al., 2015). Furthermore, they demonstrated that the phosphorylation of Ser177 preferentially recruited the E98K-OPTN to autophagosomes and enhanced autophagic flux (Sirohi et al., 2015). This led to the conclusion that the E98K-OPTN and TBK1 work together to mediate autophagic-induced cell death in RGC-5 cells. One caveat that must be mentioned when discussing these two studies is utilization of the RGC-5 cells and to what degree these findings could be translatable to neurons in the retina. The RGC-5 cell line was first published in 2001; to generate them, Ψ2 E1A virus was used to transform rat retinal ganglion cells into a cell line that could proliferate in perpetuity (Krishnamoorthy et al., 2001, 2013). After being used in more than 220 published papers, it was discovered that not only was the cell line derived from mouse instead of rat, but also that the cells were not retinal ganglion cells (Krishnamoorthy et al., 2013). Instead these cells were from a mouse transformed photoreceptor cell line known as 661 W, which were present at the same time in the laboratory that produced the RGC-5 cell line; it was thought that cross-contamination of these cell lines occurred during this time (Krishnamoorthy et al., 2013). With this in mind, it is important to understand that the studies done on the RGC-5 cell line are not a reflection of RGCs in glaucoma but can provide insight into the effects of glaucoma-associated stressors on retinal neurons. While RGCs are the most sensitive retinal cells to glaucomatous damage, there is evidence that photoreceptors and other retinal neurons may be affected. It has been suggested that this may occur as a secondary insult through a mechanism of trans-synaptic degeneration (Calkins, 2012; Zhou et al., 2019). One possible explanation for this could be the generation of oxidative stress and inflammation in the retinal environment leading to widespread stress affecting the various cells within the retina. In animal models of glaucoma and human studies, it has been observed that glaucomatous damage can lead to loss and/or swelling of cone photoreceptor cells (Nork et al., 2000; Pelzel et al., 2006; Choi et al., 2011; Ortín-Martínez et al., 2015). Electroretinogram studies have revealed functional deficits of outer retinal cells since the electrical signal is generated through the function of photoreceptors and bipolar cells, further supporting the notion that glaucomatous damage is not restricted to the RGCs and their axons (Fazio et al., 1986; Holopigian et al., 1990; Odom et al., 1990; Vaegan et al., 1995; Nork et al., 2000). Therefore, studies utilizing the RGC-5 cell can still provide important insights for investigations of glaucoma.

Lastly, there is a rare mutation that involves a 2 base pair (bp) insertion into exon 6 of the OPTN gene (691_692insAG or 2bpIns-OPTN) that is associated with NTG and amyotrophic lateral sclerosis (ALS) (Medchalmi et al., 2021). Similar to the E98K mutation, it was found that this mutation results in harmful TBK1 activation leading to impaired autophagy, ER stress, and cell death (Medchalmi et al., 2021).

## Discussion

TANK-binding kinase 1 has emerged as a critical regulator of multiple cellular processes, including inflammation, apoptosis, autophagy, and mitophagy. Its roles in the NF-κB and IRF signaling pathways, as well as its influence on mitochondrial function and cellular stress responses, establish TBK1 as a key contributor to neurodegenerative mechanisms, including in the context of glaucoma, and particularly NTG. TBK1 has been identified as a monogenic factor, with copy number variations following Mendelian inheritance patterns. These CNVs are associated with RGC apoptosis and optic nerve degeneration, underscoring TBK1’s involvement in IOP-independent pathological mechanisms. Since TBK1 has shown both pro-inflammatory and anti-inflammatory actions and there is a lack of research indicating how TBK1 duplications may affect mitophagy, there is an obvious need to continue research on this kinase in glaucoma.

Furthermore, TBK1 interacts with other critical proteins, such as OPTN, to regulate autophagic and mitophagic processes essential for maintaining neuronal health. Mutations in OPTN, such as E50K and M98K, highlight how TBK1 interactions can enhance other known genetic mutations leading to autophagic dysregulation, imbalanced neuronal homeostasis, and increased apoptosis contributing to glaucomatous neurodegeneration. Beyond glaucoma, these processes overlap with mechanisms observed in broader neurodegenerative diseases, reinforcing TBK1’s significance as a therapeutic target.

Current treatments for glaucoma focus solely on lowering IOP, yet they do not fully prevent disease progression, particularly in NTG cases where IOP appears normal. This highlights the urgent need for research into IOP-independent mechanisms of RGC stress and ON damage. TBK1’s roles in inflammation, mitophagy, and cellular stress make it a promising target for future neuroprotective strategies. Understanding TBK1’s complex contributions to glaucoma pathogenesis could pave the way for innovative therapies aimed at preserving vision and halting neurodegeneration.
